# Disseminated paracoccidioidomycosis in a patient with human immunodeficiency virus infection with the spectroscopic detection of trehalose

**DOI:** 10.1590/0037-8682-0106-2026

**Published:** 2026-06-15

**Authors:** Bruno Rigonato, Mariani Garcia de Lima, Fernanda Veloso Pereira, Monica Jacques de Moraes, Fabiano Reis

**Affiliations:** 1 Universidade Estadual de Campinas, Departamento de Radiologia e Oncologia, Campinas, SP, Brasil.; 2 Universidade Estadual de Campinas, Departamento de Medicina Interna, Campinas, SP, Brasil.

A 23-year-old male car-wash attendant with no history of rural residence or travel was admitted to the emergency department with a two-week history of persistent fever, intermittent bilateral temporal throbbing headaches, and confusion. His medical history was significant for vertically transmitted human immunodeficiency virus (HIV) infection and advanced acquired immunodeficiency syndrome (AIDS) because of low adherence to anti-retroviral therapy. Laboratory evaluation confirmed a viral load of 366,000 copies/mL and a CD4+ count of 11 cells/mm³.

**FIGURE 1:**
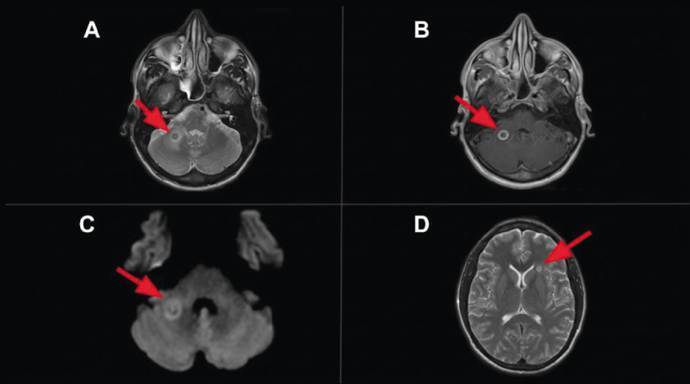
**A:** Axial T2-weighted magnetic resonance imaging (MRI) of the brain showing a well-defined intra-axial expansive lesion centered in the right cerebellar hemisphere (arrow), with a hyperintense central portion (visualized as a target-like area), and a hypointense peripheral zone. Perilesional vasogenic edema is also present. **B:** Axial T1-weighted MRI of the brain (imaged with a paramagnetic agent) showing peripheral enhancement of the lesion (arrow). **C:** Diffusion-weighted imaging showing an internal linear component and a peripheral area with restricted water diffusion (arrow). There was no diffusion restriction in the liquefied/necrotic region. **D:** Axial T2-weighted MRI of the brain showing a nodular lesion with a hyperintense periphery and hypointense center (arrow) in the left frontal periventricular white matter, but there was no perilesional edema.

**FIGURE 2:**
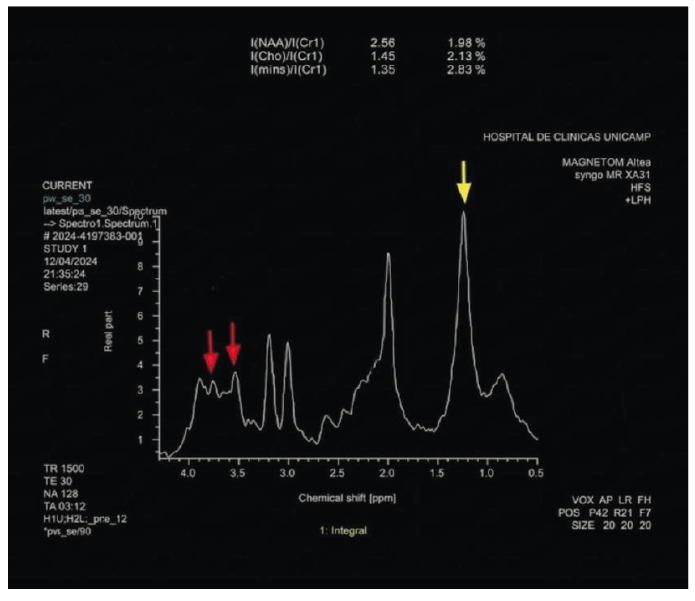
Proton magnetic resonance spectroscopy performed using a short echo time (TE; 30 ms) and a single voxel positioned in the cerebellar lesion. The analysis detected elevated lipids/lactate (at 1.3 ppm) (yellow arrow), indicative of necrosis/anerobiosis, and lipids (at 0.9 ppm), and there was no significant increase in choline (a marker of cellular proliferation at 3.2 ppm). A trehalose peak (at 3.6-3.8 ppm; red arrows), a metabolite specific for fungal infection, was also noted. These findings are consistent with a process of fungal origin.

The initial radiological assessment began with contrast-enhanced computed tomography of the cranium that revealed a focal lesion in the right cerebellar hemisphere. For a more detailed characterization, magnetic resonance imaging of the brain was performed **(** Figure 1 and Figure 2). 

The clinical history included a lymph node biopsy in which *Paracoccidioides brasiliensis* was detected. Consequently, treatment was initiated with sulfamethoxazole-trimethoprim.

**FIGURE 3:**
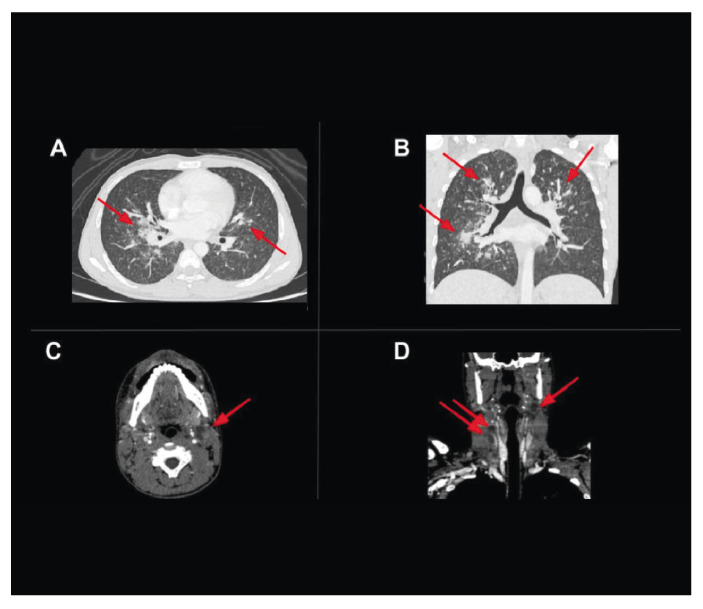
**A,B:** Axial and coronal computed tomography (CT) of the chest showing lung parenchyma with multiple, widely distributed micronodules with a miliary pattern and areas of peri-hilar consolidation (red arrows). **C,D:** Axial and coronal CT of the neck showing bilateral lymphadenopathy at multiple cervical levels, and the formation of conglomerates in some regions, with necrotic/liquefied centers (red arrows).

The diagnosis of multisystemic dissemination **(** Figure 3 **)** was definitively consolidated by a skin biopsy in which *Paracoccidioides* sp. were identified. 

Severe immunosuppression is a well-established common denominator in patients with disseminated paracoccidioidomycosis^1^. In patients with HIV/AIDS, paracoccidioidomycosis behaves as an opportunistic mycosis, leading to a higher risk of relapses and a poorer prognosis compared to immunocompetent hosts^2^. 

Proton magnetic resonance spectroscopy was useful in the diagnosis, because the detection of trehalose (peak at 3.6-3.8 ppm) is considered to be a specific metabolic marker for a fungal etiology^3^ ^,^ ^4^. This is the first report to document this specific spectroscopic finding in a patient with neuroparacoccidioidomycosis and AIDS. Identification of the fungus in samples from other regions of body (skin, cervical lymph nodes, and thorax) assisted in confirming the diagnosis^3^ ^,^ ^5^. 
